# The unhealthy-tasty intuition in dining out situations: the role of health inferences and taste expectations

**DOI:** 10.3389/fnut.2023.1152114

**Published:** 2023-05-10

**Authors:** Marion Garaus, Elisabeth Wolfsteiner, Jennifer Hu

**Affiliations:** ^1^School of International Management, Modul University Vienna, Vienna, Austria; ^2^Institute of Marketing, University of Applied Sciences Wiener Neustadt, Wiener Neustadt, Austria

**Keywords:** sensory food descriptions, healthy eating, menus, desserts, purchase intention

## Abstract

**Introduction:**

Increasing obesity rates around the globe have challenged policymakers to find strategies to prompt healthier eating habits. While unhealthy eating takes place in many different contexts, dining out is a context where individuals often choose an unhealthy option despite the availability of healthier alternatives. One possible explanation for this behavior is the unhealthy-tasty intuition, which refers to the belief that unhealthy food is tastier than healthy food. Nevertheless, many policymakers and restaurant managers follow the – in this context – counterintuitive approach of using health claims to nudge people towards more healthy eating choices or habits.

**Methods:**

The current research employs an online experiment with 137 participants and investigates how health claims and sensory claims impact on the purchase intention of healthy options for desserts. Furthermore, it explores how health inferences and taste expectations mediate the intention to purchase.

**Results and discussion:**

Findings from the online experiment confirm that health claims prompt positive health inferences, while also stimulating unfavorable taste expectations, resulting in a lower intention to purchase. Surprisingly, we found no effect of a sensory claim on taste expectations. The findings of our experiment contradict the unhealthy-tasty intuition by revealing a significant positive correlation between taste expectations and health inferences. While both health inferences and taste expectations impact positively on purchasing intentions for the health-claim condition, the indirect effect of taste expectations was stronger than the indirect effect of health inferences.

## 1. Introduction

Since the 1970s, the worldwide obesity rate has almost tripled. In 2016, 1.9 billion adults were overweight and 650 million adults were obese (1). More than half of European citizens have a body-mass index (BMI) of over 25 and so are considered overweight (2). These statistics are alarming, given that both being overweight and obese can cause severe health problems, such as heart disease, Type 2 diabetes, cancer, or strokes (3–5). In addition to these severe individual consequences, unhealthy eating practices have negative effects on society as well. The rising number of diseases caused by being overweight and obesity puts pressure on healthcare systems across the world. Interest in healthy lifestyles has grown over the last decade (6, 7), though researchers and policymakers struggle to nudge people into healthy eating habits (5). Of all the contexts in which unhealthy eating takes place, dining out is one in which it is particularly common. A study reveals that 70% of meals consumed in United States fast-food restaurants and 50% of meals offered in full-service restaurants were of poor nutritional quality. The authors conclude that “dining out is a recipe for unhealthy eating most of the time” (8). Dining out correlates significantly with energy intake, as well as the intake of fat, protein, and cholesterol (9). Howlett et al. (10) mention consumers’ increased spending at restaurants as one reason for increasing obesity rates. Consumers are generally more willing to eat unhealthy desserts rather than ones which are healthier (11). Against this background, the current research concentrates on dining out and focuses on desserts.

Eating out is acknowledged as one of Europe’s favorite leisure activities, leading to a boom in the restaurant and service industry (12). A survey of German citizens in 2017 revealed that 40% consumed meals or snacks in a restaurant at least once a week (13). Adults working full-time often consume their lunch in a canteen or restaurant close to their workplace, implying they have few opportunities to follow a healthy diet (14). Even when healthy meals are offered, consumers tend to choose the unhealthy alternative. An explanation for consumers’ preference for unhealthy food is the unhealthy-tasty intuition. Research confirms that individuals associate unhealthy food with a better taste (15), while healthy food is associated with having less taste (15, 16). Taste is one of the main predictors of food choice (17), and hence, consumers tend to neglect any health benefits in favor of unhealthy food due to the illusion that the unhealthy dish will be tastier than the alternative. However, evidence on the validity of the unhealthy-tasty intuition is inconclusive. Some research supports the unhealthy-tasty intuition [e.g., (16, 18, 19)], while some did not find any correlation or a positive correlation between healthy food and expectations of tastiness (20, 21).

These results challenge restaurant managers and policymakers since the lack of knowledge of consumers’ favorable or unfavorable associations with healthy meals hinders the adoption of the best promotion strategies. Nudging people towards healthy-eating habits has been widely researched over the last few years [see (22) for a review]. For instance, Parkin and Attwood (23) investigated menu composition and the impact of vegetarian symbols. Their study reveals that a minimum of 75% of dishes need to be vegetarian to prompt diners to choose a vegetarian dish, while a vegetarian symbol did not impact on choice. Other research confirms that appealing food descriptors can increase the popularity of vegetarian dishes (24–26). Despite this knowledge, several restaurants often rely on a different strategy, namely communicating the health benefits of dishes in the form of health claims. This practice is supported by research providing evidence of the positive effect of favorable nutrition information on nutrition attitude and the intention to purchase (27). Likewise, research indicates the positive effect of taste labels on the favorable taste expectations of healthy food (24). In contrast, other studies report the negative effect of health claims on consumer responses. For instance, a reduced-fat label decreased the liking for chocolate compared to a condition in which there was no label (28). The inconclusive findings on the effectiveness of health claims in nudging people into healthy eating habits make it difficult for restaurant managers to optimize their menus, as well as for policymakers to promote the choice of healthy food. The current study seeks to add to the discussion on the effectiveness of health claims in the context of restaurant menus. Additionally, another objective of this study is to test the so far under-researched claims on restaurant menus when promoting healthy food choices, namely sensory claims. Sensory claims appeal to an individual’s senses through verbal descriptions, for instance, the word “crunchy” communicates information about the texture of a dish, while the word “sweet” might prompt expectations of taste. Sensory claims can prompt taste expectations and can affect overall product evaluations positively (29). However, there is limited knowledge of the role of sensory claims in promoting the intention to purchase healthy food in the context of dining out. Furthermore, it seems that healthy dishes are described in less appealing words in general (30).

Hence, the current study seeks to shed some light on the effectiveness of health claims on restaurant menus, while at the same time testing a novel alternative for prompting healthy eating, namely sensory claims. From a theoretical perspective, the major objective of this research is to investigate the importance of using health claims on menus. Additionally, we seek to offer additional insights into the effectiveness of an alternative way of describing dishes on menus on the intention to purchase food, using sensorially descriptive food names in a restaurant setting. Finally, another objective of this study is to test the unhealthy-tasty intuition in the context of dining out with a sample of people from the United Kingdom.

The findings of this study contribute to the existing literature in at least three ways. First, we contribute to the ongoing debate on the prevalence of the unhealthy-tasty intuition. In doing so, we test if health claims as food cues activate the unhealthy-tasty intuition which results in increased healthiness perceptions but lower taste expectations. Second, we investigate how healthiness perceptions are directly related to taste expectations. In other words—and in contrast to existing studies—we investigate how the two mediating processes health inferences and taste expectations relate to each other and if health inferences impact taste expectations positively or negatively. Third, we do not only explore how health claims stimulate healthiness expectations but also how a sensory claim in the form of taste cues prompts health inferences and taste expectations. Hence, we expand existing literature that concentrates on practices to nudge healthy eating habits [e.g., (24)], while concentrating on a specific type of food descriptors, namely sensory food descriptors. Although other research has concentrated on appealing food descriptors in the past, the role of sensory food descriptors (i.e., words that appeal to a food’s taste or texture) is under researched.

The following section provides an overview of the unhealthy-tasty intuition, the theoretical framework for the current research. Furthermore, it reviews the extant studies on the impact of health claims on the choice of healthy food, followed by an explanation of the relationship between taste expectations and health inferences. The literature review concludes with a review of the effects of sensory claims on the choice of healthy food. It is followed by a description of the design, analysis, and results of an online experiment. The paper concludes with a discussion of the theoretical and practical contributions of the results and suggests avenues for future research.

## 2. Literature review

### 2.1. The unhealthy-tasty intuition

The unhealthy-tasty intuition claims that consumers associate unhealthy food with tasting better compared to healthy food, leading to lower consumption of the latter (15). Compelling evidence exists for a positive correlation between a consumer’s unhealthy-tasty belief and an individual’s BMI (5, 31). More specifically, Cooremans et al. (31) report that the unhealthy-tasty intuition increases obesity rates by 1.18 times for consumers in the United States, United Kingdom, France, and Belgium. Results from two surveys conducted in Germany confirm that a stronger unhealthy-tasty belief is associated with a higher BMI (5). Another cross-sectional survey conducted in Australia, Germany, Hong Kong, India, and the United Kingdom validates these results: a stronger unhealthy-tasty belief correlates positively with BMI, and this relationship is mediated through a lower consumption of fruits and vegetables (32).

Several theoretical explanations for the unhealthy-tasty intuition have been put forward in the extant literature. From an evolutionary perspective, food high in sugar, and fat, or energy-dense products in general, are vital for survival and hence comprises an evolutionary advantage (33). Other studies refer to the loss of freedom and choice derived from limiting unhealthy consumption during childhood. Many parents try to prevent their children from consuming unhealthy (but tastier) food while at the same time fostering the consumption of healthy (but less tasty) food. Discouraging consumers from eating unhealthy food can cause a reinforcement effect through a loss of freedom and choice (15). Once consumers have a choice, they are more likely to choose the unhealthy alternative due to a lack of self-control, especially when pursuing two conflicting goals, i.e., the choice of healthy vs. tasty food (5). In other words, the unhealthy-tasty intuition suggests that it is difficult to satisfy both taste and health considerations, and the satisfaction of the latter prevents the satisfaction of taste (15). Consequently, consumers face the challenge of balancing short-term and long-term consequences when making their food choices. A tasty (and unhealthy) alternative causes short-term pleasure, while the long-term health benefits appear less concrete (34). Consumers tend to choose short-term pleasure over long-term health benefits, and this seems to be particularly prevalent when choosing desserts (11).

Despite the compelling evidence for, and the theoretical reasoning behind, the unhealthy-tasty intuition, some studies report the opposite effect, i.e., the positive effect of health inferences on taste expectations. Methodologically, these studies differ from the research confirming the unhealthy-tasty intuition by asking respondents to rate products on two separate scales assessing healthiness and tastiness, rather than explicitly measuring the unhealthy-tasty intuition [e.g., (15)]. The study of Werle et al. (35) reports a positive relationship between healthiness and taste for French consumers measured with an implicit association test. Accordingly, cultural differences need to be considered when investigating the unhealthy-tasty intuition. Following this suggestion, Haasova and Florack (21) confirmed that the unhealthy-tasty intuition does not hold in the context of fast-moving consumer goods (FMCG). The authors presented respondents with 20 snacks and 20 soft drinks and asked them to rate these products for healthiness and tastiness using two different rating scales. In two studies, Austrian and German consumers associated healthy products with a good taste. Findings from Werle et al. (35) and Haasova and Florack (21) acknowledge the relevance of cultural differences when exploring the unhealthy-tasty intuition.

The food-pleasure orientation might explain these cultural differences. This describes the extent to which individuals associate food consumption with sensorial and social pleasure, instead of associations with its consequences for health and utilitarian effects (36). France is considered to be a highly food-pleasure-oriented country (36) and research confirms that individuals with low pleasure orientation evaluate healthy food as being less tasty and are more likely to choose an unhealthy dessert after they have had a healthy main dish (3). Hence, food-pleasure orientation among cultures seems to diminish the unhealthy-tasty orientation (3). Other research reports that both US consumers and Indian consumers associated healthiness with tastiness, while this effect was negatively moderated by the level of processing: A higher level of processing (of pulses) attenuated the association between health inferences and taste expectations for US respondents (20).

However, not only cultural differences but also the rising amount of information on the consequences of unhealthy eating might educate consumers and hence counteract the unhealthy-tasty intuition. Indeed, rising obesity rates have prompted the intensification of educational measures to combat the prevailing assumption that healthy food is less tasty (37). For instance, the United Kingdom government introduced the *Eatwell Guide* in 2016 (38) together with dietary restrictions (39). A survey from the British Nutrition Foundation (40) reports that 62% of respondents changed their diet to a healthier one in 2020/2021. As a consequence, many brands seek to associate their products with a healthy image, while at the same time promising that they will be tasty (21). Taste is then one of the most important food values and a decisive factor for food choice. The next section describes how different claims (health claims, sensory claims) are expected to impact on health inferences and taste expectations.

### 2.2. Nudging healthy food choices

Consumers are often confronted with incomplete information during the choice of food (41). One way to compensate for this lack of information is to rely on food cues. Product-related cues prompt expectations which in turn can impact on the experience (42). This occurs especially when consumers cannot judge food products based on their sensory properties (e.g., smell, taste)—usually from prior consumption (43)—and they have to rely on extrinsic food cues (42). Extrinsic food cues are characteristics that are not physically part of the product (44) and hence can be amended without changing the product itself (43). One very common way of presenting product-relevant information is labeling, especially in the context of fast-moving consumer goods (42). The impact of food labels has received considerable research attention in the context of fast-moving consumer goods [e.g., (27, 45–47)]. However, food labels are also commonly used in a hospitality context where restaurant menus try to prompt food choices by adding labels such as “suggestion of the chef” (48). Given the increasing trend towards health awareness (49), several products rely on labels to offer consumers options for a healthy diet.

Extant research acknowledges the relevance of health claims in restaurant menu design. For instance, Kozup et al. (27) claim that “the substantial number of nutrient and health claims appearing on both restaurant menus and packaged food labels highlights the importance of understanding how consumers use health claims.” Likewise, a study explored frequently used nutrition claims (e.g., low fat, low energy, low sugar, or light) on menus and reported that after sensory attributes, nutritional properties represent the second-most interesting labels (50). Nevertheless, evidence suggests that health claims are rather ineffective in nudging people towards healthy eating. An eye-tracking study with FMCG as stimuli reports that a health label does not impact on the choice of food (51). Confirming this finding, health warning labels do not impact on the choice of food, although they negatively affect brand attitude (52). Likewise, claims of weight reduction through the consumption of whole grains did not affect food choices (53). Even worse, and in confirmation of the unhealthy-tasty intuition, some studies report the negative effect of health claims on consumers’ taste expectations. Since taste is one of the most important predictors of food choice [Howlett et al., 2009 (10)], diminishing taste expectations because of health claims might impact negatively on the choice of food. Although the empirical evidence on the unhealthy-tasty intuition is rather inconclusive, it does exist for the negative effect of health claims on taste expectations. In an eating-out context especially, it is reasonable to assume that short-term benefits (i.e., taste) might outperform long-term benefits (i.e., health). Some empirical evidence supports these notions. A reduced-fat label decreased the liking of chocolate when compared to a non-label condition (28). Another study conducted with FMCG reported similar results: a claim of health benefits decreased the taste experience and resulted in a decreased intention to purchase (16). Likewise, a recipe which was labeled as healthy reduced taste expectations and the intention to cook compared to one which was not labeled (54). In a field study, participants reported lower attitudes and intentions to purchase towards products that were labeled as being low-calorie together with the actual calories of the item [Howlett et al., 2009 (10)]. Following this, we suggest that:

*H1*: Health claims negatively affect taste expectations.

*H2*: Health claims negatively affect the intention to purchase through taste expectations.

Nevertheless, a health claim seems to be effective in promoting health inferences (54, 55). Making health inferences requires consumers to understand a health claim, and hence requires a degree of cognitive effort (56). More specifically, the spreading activation model suggests that if a specific node in a human memory structure is activated (e.g., through a health claim), additional nodes and concepts related to this network might be activated as well (57). It seems that the elaboration of health claims and associated health benefits results in favorable consumer responses. Despite the negative impact of health claims on taste expectations observed in the study of Garaus and Lalicic (54), the authors report a positive indirect effect of health claims on behavior intentions mediated by health inferences. Other research also demonstrates the positive effect of health claims. For instance, Kozup et al. (27) show the positive effect of health claims and nutrition information on the attitude towards the product and the intention to purchase.

*H3*: Health claims positively affect health inferences.

*H4*: Health claims positively affect the intention to purchase through health inferences.

Even when health claims can prompt favorable health inferences, which in turn prompt the intention to purchase, it seems that this positive effect is canceled out by the negative indirect effect of taste expectations [see (54)]. Nevertheless, one way to overcome the ineffectiveness of health claims in promoting healthy behavior might be the stimulation of taste expectations via health inferences. Despite the prevalence of the unhealthy-tasty intuition, studies measuring taste expectations and health inferences on separate scales (in contrast to studies that directly ask respondents if they associated healthy food with having less taste) report a positive correlation between perceptions of healthiness and tastiness (20, 21, 35). In support of this, a recent study showed that organic labeling prompts both, health inferences and favorable taste expectations and that the positive effect on taste expectations is mediated by health inferences (58). Therefore, we propose that:

*H5*: Health claims positively affect intention to purchase if the favorable health inferences prompt favorable taste expectations.

In addition to health claims, the current study seeks to test the effectiveness of sensory claims. One category of food descriptors is sensory food descriptors. This type of descriptive naming offers insights into the sensory properties of food. In contrast to food choices made in a grocery store, consumers cannot see, smell or touch dishes before they are ordered. Accordingly, consumers have to rely on descriptors on a menu which refer to the sensory properties of a dish (59). Sensory descriptors have been found to be a signal of quality for fruits, vegetables, and wine (60). Since taste is decisive in making food choices [Howlett et al., 2009 (10)], sensory descriptors of how a dish tastes might stimulate food choice. Indeed, sensory properties are an important determinant of the consumption of vegetables (61). Past behavior seems to influence the effectiveness of sensory words on restaurant menus in terms of prompting specific food choices. An online experiment revealed that using descriptive words (i.e., “fresh and seasonal”) increased the choice of a vegetarian option for those who would rarely eat such a dish, but reduced it for those who frequently chose one (26). Another recent study reports a significant increase in healthy food choices for items which were labeled as being tasty (e.g., mouthwatering, indulgent) in a series of real-world experiments (62). However, some studies investigating the responses to sensory claims of specific target groups do not report any effect of food descriptors on food choice. In a field study conducted in Canada, descriptive food names (e.g., wonderful, creamy, wacky) did not impact on children’s choices of healthy food items (63). Likewise, no effect was found for descriptive food names among people aged 60 + in the United States. In this study, healthy dishes were manipulated by visual highlights (i.e., boxes) and enticing language (e.g., organic, gently steamed, flavorful, seasoned) (64). However, since these target groups are not the focus of this study (i.e., dining out), based on extant literature it can be concluded that sensory attributes might prompt favorable taste expectations and, hence, positively impact on the intention to purchase.

*H6*: Sensory claims positively affect taste expectations.

*H7*: Sensory claims positively affect the intention to purchase through taste expectations.

**Figure 1:**
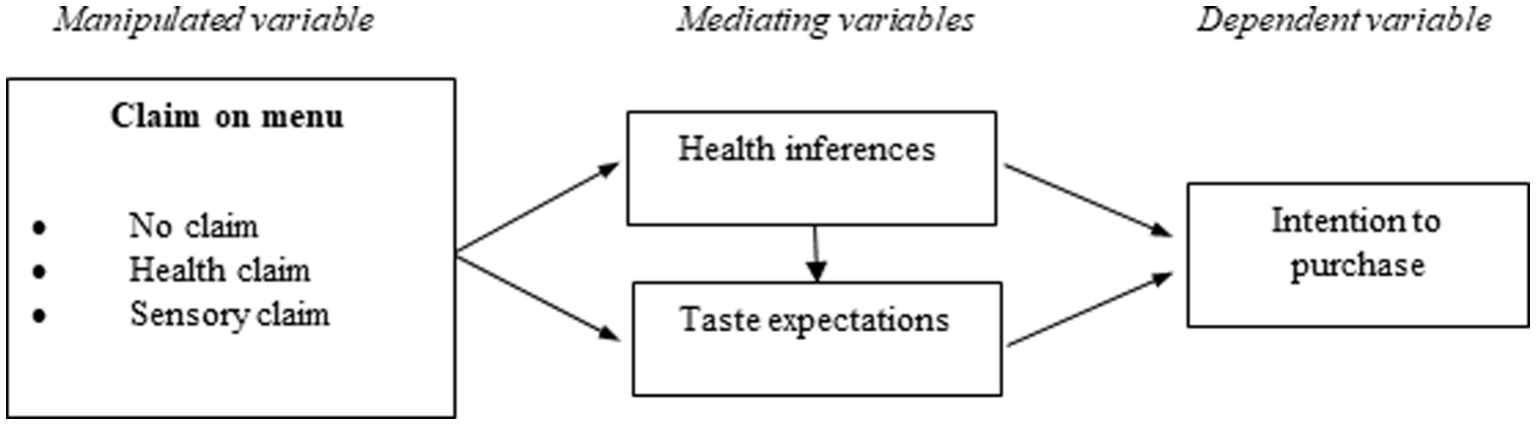
Conceptual research framework.

Figure 1 illustrates the conceptual research framework.

## 3. Materials and methods

### 3.1. Participants and design

The study employed a one-factor, between-subjects design with claims (no claim vs. health claim vs. sensory claim) representing the manipulated variable. An *a priori* power analysis (65) for mean comparisons of three experimental groups and three covariates suggested a required sample size of 251 participants (*p* = 0.05, effect size: 0.25). To assure sufficient power, we aimed for 270 respondents. Therefore, a sample of 273 participants was randomly allocated to one of the three experimental conditions. Respondents were recruited through the platform clickworker.com and were paid 50 cents for participating. English-speaking respondents living in the United Kingdom aged between 18 and 65 years qualified for the survey. The survey was completed on a desktop or notebook. The survey duration was 3–4 min and the data were collected on December 16, 2022.

The experiment included attention checks (“Please tick the middle of the scale,” “Please let us know which of the following dessert was NOT included in the menu”) and excluded those who failed the checks (88 participants). Additionally, respondents who indicated that they follow dietary restrictions which were relevant to the choice of dessert were not considered for further analysis (48 respondents). Following this, the final sample consisted of 137 participants (45% female) with a mean age of 38.45 years (*SD* = 10.10). This sample size was considered as sufficient to detect an effect of small to moderate size (0.35) at a significance level of 0.95, as yielded by another power analysis. Participants varied according to their highest level of education: 64% had completed university, 26% had finished high school, 2% had an apprenticeship, 7% had completed vocational school, and 1% had attended compulsory school. The mean value for healthy eating habits was 4.25 (*SD* = 1.07), which does not only signify a rather high relevance of healthy eating habits but also might indicate high levels of environmental consciousness given the acknowledged correlation between healthy eating habits and environmental consciousness (66) as well as the positive correlation between healthy eating habits and positive attitude towards green products (67). Table 1 summarizes the demographic characteristics (age, gender, education, healthy eating habits) of the final sample divided into the three experimental groups.

**Table 1:** Summary of age, gender, education, and healthy eating habits.

	Age	Gender	Education	Healthy eating habits
No-claim group (*N* = 41)	*M* = 38.93 *SD* = 9.62	39% female 61% male	University degree: 61%	*M* = 4.08 *SD* = 0.96
			High school: 29%	
			Apprenticeship: 2%	
			Vocational school: 8%	
			Compulsory school: 0%	
Health claim (*N* = 45)	*M* = 38.16 *SD* = 10.49	36% female 64% male	University degree: 64%	*M* = 4.37 *SD* = 1.11
			High school: 31%	
			Apprenticeship: 0%	
			Vocational school: 5%	
			Compulsory school: 0%	
Sensory claim (*N* = 51)	*M* = 38.33 *SD* = 10.31	59% female 41% male	University degree: 67%	*M* = 4.28 *SD* = 1.13
			High school: 20%	
			Apprenticeship: 2%	
			Vocational school: 10%	
			Compulsory school: 2%	
Total (*N* = 137)	*M* = 38.45 *SD* = 10.10	45% female 54% male	University degree: 64%	*M* = 4.25 *SD* = 1.07
			High school: 26%	
			Apprenticeship: 2%	
			Vocational school: 7%	
			Compulsory school: 1%	

### 3.2. Procedure

**Figure 2:**
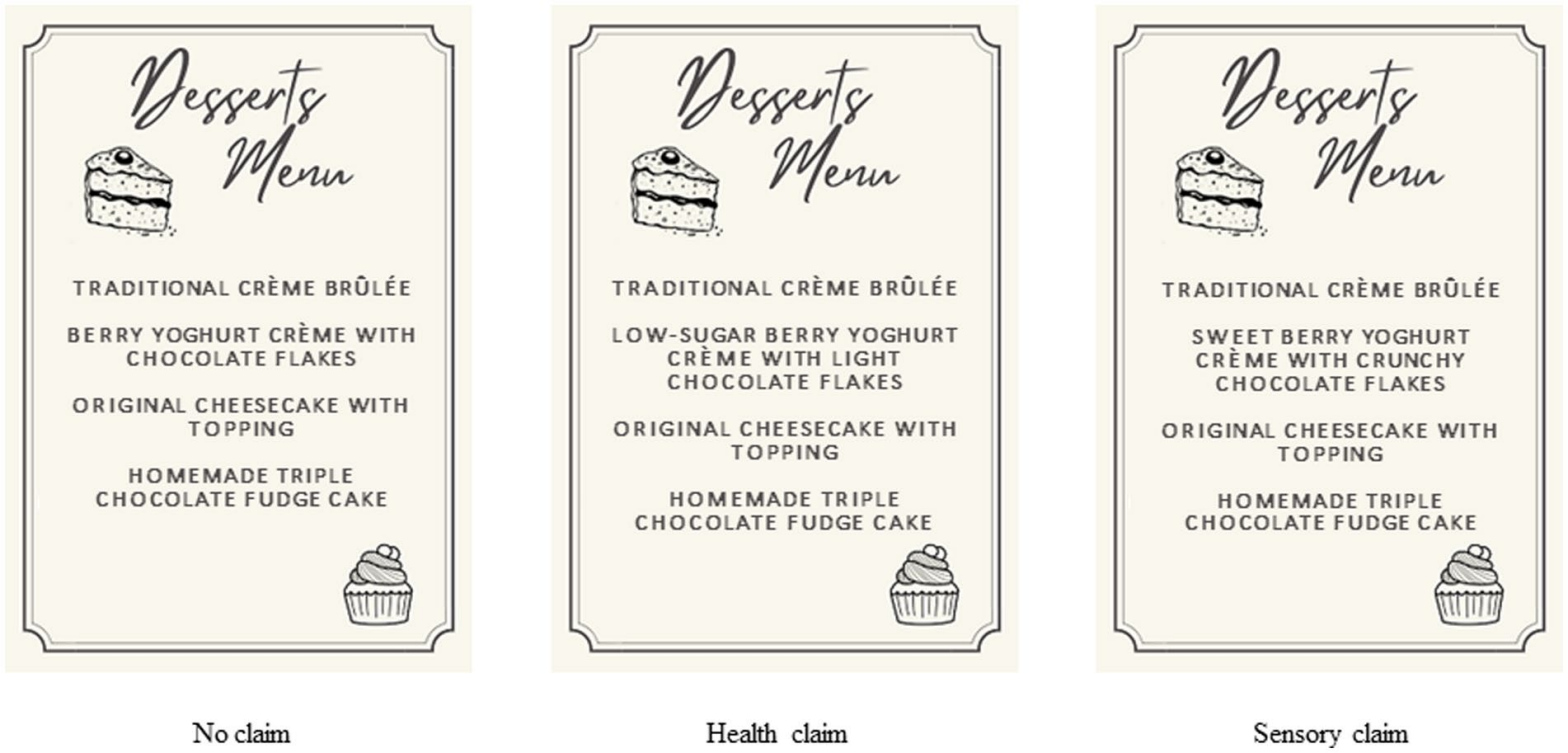
Stimulus material.

Fictitious menus including four different desserts served as stimuli. To ensure a high degree of external validity of our stimuli, we screened existing real-world menus which guided the selection of dishes on the menu card. Of the desserts, one was the manipulated dish. The dishes were: a traditional crème brûlée, berry yogurt crème with chocolate flakes, an original cheesecake with topping, and a homemade triple-chocolate fudge cake. The berry yogurt crème with chocolate flakes represented the healthy target dish and was manipulated by adding a health or sensory claim. In the health condition, “low sugar” and “light” were added, since sugar content is strongly associated with the perception of healthiness due to its direct influence on calorie intake (14). Therefore, respondents in the health-claim condition were exposed to the low sugar and berry yogurt crème with light chocolate flakes. In the sensory condition, “sweet” and “crunchy” were added since both texture and taste attributes strongly impact on taste perceptions (68, 69). Consequently, respondents in the sensory-claim condition saw the sweet berry yogurt crème with crunchy chocolate flakes on their menu (see Figure 2). All other dishes remained the same under all conditions, only the target dish was manipulated. To account for order effects, the appearance of the target dish on the menus varied systematically in all three conditions. In other words, the target dish was placed as the second dish on half the stimulus material, while it appeared as the third dish on the others. In all conditions, the menu was presented for a minimum duration of 20 s. After exposure to the stimulus, participants were asked to complete the online questionnaire.

### 3.3. Measures

The questionnaire started with three items (“Some dishes of this menu included information about the healthiness (e.g., fat content, sugar content, nutritional value),” “Some dishes of this menu included information about the taste (e.g., sour, sweet, salty),” “Some dishes of this menu included information about the consistency (e.g., crispy, crunchy, fried)”) for a manipulation check. Whether the manipulation worked as intended was assessed on seven-point Likert scales (1-strongly disagree to 7-strongly agree).

The respondents were then exposed to the target dish again and were asked to answer questions related to health inferences, taste expectations, and intention to purchase. To assess the two underlying mechanism health inferences and taste expectations, we followed the approach of Haasova and Florack (21) and measured the unhealthy-tasty intuition on two separate scales. The three items for health inferences (measured on a seven-point Likert scale; 1-strongly disagree to 7-strongly agree) were based on the study of Huang and Lu (70) and read as follows: “This dessert is part of a healthy diet,” “This dessert is healthy for me,” and “This dessert is nutritious.” The two items for the taste-expectation measure (a seven-point Likert scale; 1-not at all to 7-very) were adopted from Raghunathan et al. (15): “How much would you enjoy eating this dessert?” and “How tasty do you think this dessert would be?” Three items based on Bialkova et al. (16) assessed the intention to purchase with a seven-point Likert scale (1-strongly disagree to 7-strongly agree): “The probability that I would consider ordering this dish is high,” “I would like to recommend this dish to my friends,” and “The likelihood of ordering this dish is high.” Finally, we included six items adapted from the health motivation construct of Roininen et al. (71) to control for healthy eating habits. The items (measured on a seven-point Likert scale: 1-strongly disagree to 7-strongly agree) were: “I try to eat nutritiously,” “The healthiness of food has little impact on my food choices,” “The healthiness of snacks makes no difference to me,” “I eat what I like and I do not worry about the healthiness of food,” “I carefully watch what I eat,” and “I always follow a healthy and balanced diet.”

We calculated composite scores for health inferences, taste expectations, intention to purchase, and healthy eating habits; mean values were used for a serial multi-categorical mediation model. Table 2 summarizes the measurement of all the constructs.

**Table 2:** Measurement of constructs.

Construct/items	Cronbach’s alpha/Correlation
Health inferences (70)	0.90
*7-point Likert scale (strongly disagree–strongly agree)*	
This dessert is part of a healthy diet.	
This dessert is healthy for me.	
This dessert is nutritious.	
Taste expectations (15)	0.84
*7-point scale (not at all–very)*	
How much would you enjoy eating this dessert?	
How tasty do you think this dessert would be?	
Purchase intention (16)	0.94
*7-point Likert scale (strongly disagree–strongly agree)*	
The probability that I would consider ordering this dish is high.	
I would like to recommend this dish to my friends.	
The likelihood of ordering this dish is high.	
Healthy eating habits (71)	0.77
*7-point Likert scale (strongly disagree–strongly agree)*	
I try to eat nutritiously	
The healthiness of food has little impact on my food choices^*^	
The healthiness of snacks makes no difference to me^*^	
I eat what I like and I do not worry about the healthiness of food^*^	
I carefully watch what I eat	
I always follow a healthy and balanced diet	

^*^Items were reverse coded.

### 3.4. Preliminary data analysis

The analysis started with the investigation of the effectiveness of the manipulation. A multivariate analysis of variance (MANOVA) with the experimental condition as a factor variable and the health-claim- and sensory-claim-manipulation measures as dependent measures confirmed that the manipulation of our stimulus material was successful. The analysis revealed a significant model (Pillai’s trace: 0.28, *F*(4, 268) = 10.76, *p* < 0.01). Respondents in the health-claim condition agreed that health information was used more often to describe some dishes compared to the other conditions (*F*(2, 134) = 10.22, *p* < 0.01, *M*_health_ = 3.71 vs. *M*_control_ = 2.17 vs. *M*_sensory_ = 1.98). In contrast, respondents exposed to the sensory claim showed a stronger agreement that information about taste and consistency was used compared to respondents from the health claim and control condition (*F*(2, 134) = 9.29, *p* < 0.01, *M*_sensory_ = 4.74 vs. *M*_health_ = 4.03 vs. *M*_control_ = 3.26). We further assessed if participants in the three conditions differ in terms of healthy eating habits, since a significant difference might bias the results. An ANOVA revealed no significant differences between the three experimental groups (*F*(2, 134) = 0.82, *p* = 0.44, *M*_control_ = 4.08 vs. *M*_health_ = 4.37 vs. *M*_sensory_ = 4.28).

## 4. Analysis and results

The analysis continued with the testing of the hypotheses. A serial multi-categorical mediation model (model 6, 5.000 bootstrap samples, 95% CI) using the PROCESS macro for SPSS (72) tested H1–H7. Dummy coding was necessary for the independent variable in the multi-categorical mediation model: the control group was coded as 0, the health-claim condition was coded as 1 and the sensory-claim condition was coded as 2. The control group (the no-claim condition) served as the reference category, consequently the health-claim condition and the sensory-claim condition are compared to the no-claim condition. All effects must be interpreted in comparison to the control group. Health inferences were specified as the first mediator, taste expectations represented the second mediator, and the intention to purchase was included as the dependent variable in the model. Additionally, healthy eating habits, age, and gender served as covariates.

The inspection of the direct effect of the manipulated health claim on taste expectation confirmed H1. Results showed a significant negative effect of the health claim on taste expectations (*a_3_* = −0.65, *p* = 0.04) when compared to the control condition with no claim. Additionally, taste expectations significantly increased the intention to purchase (*b_2_* = 0.83, *p* < 0.01). Results confirmed the negative indirect effect postulated in H2 (health claims → taste expectations → purchase intention): Taste expectations mediate the negative influence of a health claim (vs. no claim) on the intention to purchase (*a_3_b_2_* = −0.53, *95% CI*[−1.04, −0.05]).

In corroboration of H3, the health claim had a significant positive effect on health inferences (*a_1_* = 0.93, *p* = 0.01) when compared to the no-claim condition. Health inferences increased the intention to purchase (*b_1_* = 0.25, *p* < 0.01). The indirect effect of the health-claim condition (vs. no-claim condition) on the intention to purchase (health claims → health inferences → purchase intention) was significant (*a_1_b_1_* = 0.23, *95% CI*[0.06, 0.49]). Hence, H4 was supported. In H5 we postulated an indirect effect of the health claim (vs. no claim) on the intention to purchase through health inferences and taste expectations (health claim → health inferences → taste expectations → purchase intention). Health inferences increased taste expectations (*d_21_* = 0.31, *p* < 0.01). As already discussed, a health claim had a positive effect on health inferences (see H3) and taste expectations increased the intention to purchase (see the results for H2). Taken together, the results pointed to the mediating effect of the health inferences and taste expectations of a health claim on the intention to purchase (*a_1_d_21_b_2_* = 0.24, *95% CI*[0.06, 0.47]).

**Figure 3:**
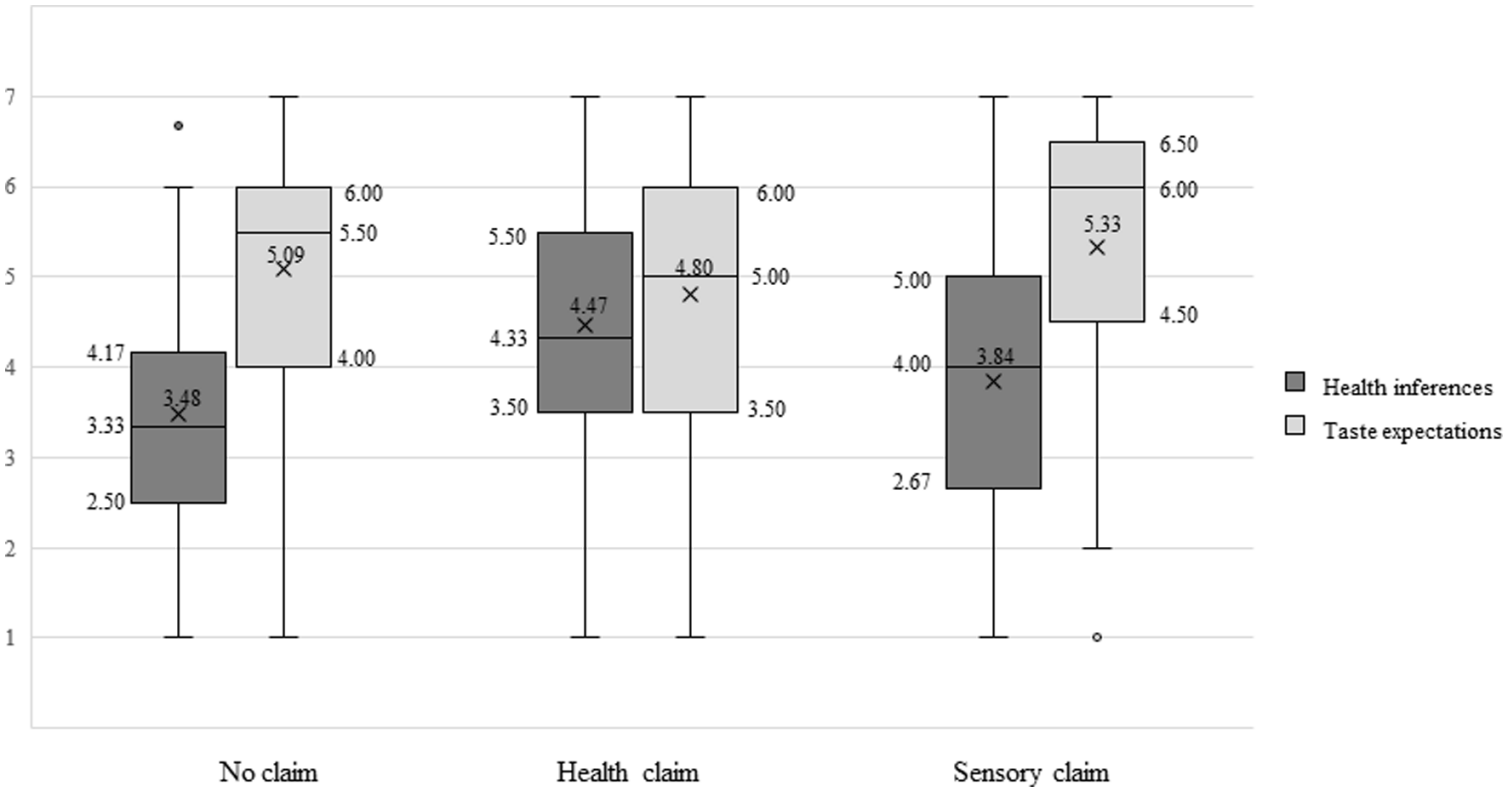
Box plots of the distributions of health inferences and taste expectations in the no claim, health claim, and sensory claim condition.

In contrast to H6, a sensory claim did not increase taste expectations (*a_4_* = 0.25, *p* = 0.41) when compared to the control condition with no claim. Consequently, the indirect effect of the sensory-claim condition on the intention to purchase (sensory claim → taste expectations → purchase intention) was not significant (*a_4_b_2_* = 0.21, *95% CI*[−0.32, 0.75]); hence H7 was not supported. Table 3 summarizes the results of the multi-categorical serial mediation model and Figure 3 visualizes the minimum/maximum, the first/third quartile, and the median/mean value for health inferences and taste expectations for the three experimental conditions.

**Table 3:** Relative direct and indirect effects.

Variable	M_1_ Health inferences				M_2_ Taste expectations				Y_1_ Purchase intention			
	Coeff.	*SE*	*p*		Coeff.	*SE*	*p*		Coeff.	*SE*	*p*
Health claim	*a_1_*	0.93	0.34	0.01	*a_3_*	−0.65	0.32	0.04	*c´_1_*	−0.74	0.24	0.00
Sensory claim	*a_2_*	0.31	0.33	0.35	*a_4_*	0.25	0.30	0.41	*c´_2_*	−0.27	0.22	0.22
Health inferences (M_1_)					*d_21_*	0.31	0.08	0.00	*b_1_*	0.25	0.06	0.00
Taste expectations (*M_2_*)									*b_2_*	0.83	0.07	0.00
Healthy eating habits (Cov)	*f_1_*	0.17	0.12	0.17	*g_1_*	0.03	0.11	0.81	*h_1_*	−0.12	0.09	0.16
Age (Cov)	*f_2_*	−0.01	0.01	0.59	*g_2_*	−0.03	0.01	0.02	*h_2_*	−0.00	0.01	0.62
Gender (Cov)	*f_3_*	0.09	0.28	0.75	*g_3_*	−0.71	0.25	0.01	*h_3_*	−0.36	0.19	0.07
Constant	*i_M1_*	2.92	0.90	0.00	*i_M2_*	5.97	0.86	0.00	*i_y_*	0.53	0.74	0.47
	*R*^2^ = 0.08				*R*^2^ = 0.19				*R*^2^ = 0.68			
	*F*(5,131) = 2.26, *p* = 0.05				*F*(6,130) = 5.07, *p* < 0.01				*F*(7,129) = 39.61, *p* < 0.01			

									Coeff.	CI		
*Relative indirect effects (health inferences)*												
Health claim							*a_1_b_1_*		0.23	[0.06, 0.49]		
Sensory claim							*a_2_b_1_*		0.08	[−0.08, 0.26]		
			
*Relative indirect effects (taste expectations)*												
Health claim							*a_3_b_2_*		−0.53	[−1.04, −0.05]		
Sensory claim							*a_4_b_2_*		0.21	[−0.32, 0.75]		
			
*Relative indirect effects (health inferences → taste expectations)*												
Health claim							*a_1_d_21_b_2_*		0.24	[0.06, 0.47]		
Sensory claim							*a_2_d_21_b_2_*		0.08	[−0.08, 0.26]		

## 5. Discussion

Given the alarming obesity rates around the globe, policymakers need to find new ways to guide citizens towards a healthy lifestyle. While several measures are currently in place, such as the development of guidelines on healthy-eating practices [e.g., (38, 39)], the choice of unhealthy food while dining out has received only limited attention. This is surprising as there is general agreement that dining out is associated with unhealthy eating (8–10). In contrast to prior research, the current research investigates the effect of health claims in the context of dining out. Due the increasing trend of consuming meals and snacks in restaurants (12) and its associated unhealthy eating habits (9, 10) this particular context should not be neglected when guiding societies towards healthy lifestyles, and one possible explanation for unhealthy eating practices is the unhealthy-tasty intuition (5, 15, 31).

Drawing on the unhealthy-tasty intuition, the current research challenges the practice of relying on health claims on menus to prompt healthy food choices. Results from an online experiment reveal that health claims reduce taste expectations, which results in lower levels of intention to purchase. This result corroborates prior studies reporting a negative relationship between health claims and taste expectations [e.g., (28, 54)]. While health claims decrease taste expectations, at the same time they also prompt favorable health inferences, which in turn have a positive effect on the intention to purchase. Nevertheless, a significant negative direct effect (*c^´^_1_* = −0.74, *p* < 0.01) and a significant negative total effect (−0.80, *p* = 0.04) reveal that the negative indirect impact of taste expectations is stronger than the positive indirect impact of health inferences. Although research acknowledges the relevance of both health and sensory characteristics (i.e., taste) in predicting food choice (44), several studies confirm the superior role of the taste of food in decision-making (15, 73).

Ideally, healthy food should prompt both health inferences and favorable taste expectations. As the findings of our study reveal, health claims have a direct negative impact on taste expectations, however, health inferences and taste expectations correlate positively. In other words, the health inferences derived from a health claim can prompt positive taste expectations, which in turn increase the intention to purchase. Accordingly, health inferences not only impact directly on the intention to purchase, but also indirectly through favorable taste expectations. It can be concluded, therefore, that the negative effect of health claims might be attenuated if claims can stimulate the cognitive processing of health claims enabling individuals to draw health inferences. Accordingly, from a theoretical perspective, the findings of our study highlight the importance of prompting consumers to consider a health claim: If consumers do not think about the benefits associated with the health claim (i.e., health inference), they might intuitively evaluate the expected taste first, which is significantly lower compared to a condition where there is no label. On the contrary, if consumers deliberately think about the health benefits associated with a healthy dish (promoted by a health claim), these health inferences further prompt favorable taste expectations. However, this effect might only work for food which is inherently healthful, since this type of food is associated with having a good taste (74). In this regard, it needs to be noted that the sequence of measuring might have affected the result. We prompted participants to first make health inferences before we asked respondents to indicate their taste expectations. To avoid any sequence effects, future studies that replicate our results might consider a systemic variation of the measurement of these two constructs. Then, it could be explored if the indirect link through health inferences also holds when taste expectations are measured before health inferences, which would further strengthen the validity of our results.

This finding is particularly interesting since it contradicts the unhealthy-tasty intuition, which postulates that individuals associate unhealthy food with having a better taste (5, 15, 31). Nevertheless, our findings replicate existing research employing an indirect measurement of the unhealthy-tasty intuition by measuring health inferences and taste expectations on two separate scales [e.g., (21)]. Hence, it seems that the validity of the unhealthy-tasty intuition mainly depends on the approach taken towards measurement, although this requires further investigation.

Surprisingly, we did not observe any effect of a sensory claim on taste expectations. Although the sensory claim increased taste expectations compared to the health claim, no significant difference was observed between the no-claim condition and the sensory-claim condition. This result contradicts previous studies reporting a positive effect of taste claims on healthy food choice [e.g., (26, 62)] but replicates studies that concentrate on the responses of elderly people or children to sensory claims (63, 64). One possible explanation for the inconsistent findings in extant literature is the highly fragmented terminology of sensory attributes. While we used specific taste claims (sweet and crunchy) other studies that report a positive effect of taste claims on food choices often rely on hedonic descriptors [e.g., (75)] or indulgent labels [e.g., mouthwatering, (24)], which are more appealing and diagnostic than taste claims. In general, using the correct words for describing menus is acknowledged to be a challenging task. Jurafsky (76) claims that some food descriptors can be considered as “linguistic fillers” such as the word “delicious” that is quite vague and provides little informative value. While appealing adjectives (e.g., crispy or crunchy) provide more informative value, the author challenge its effectiveness in prompting food choices due to the highly subjective nature (76). This reasoning might explain why sensory claims are not always effective, and based on our findings, they do not offer a good alternative way of promoting healthy food choice. Nevertheless, the nature of the stimulus material might explain this unexpected finding: An inspection of the means suggests that the dessert seems to prompt relatively high taste expectations even without a sensory claim (*M*_taste_control_ = 5.09). Given this strong favorable taste association, it is difficult to further enhance taste expectations with additional information, causing a ceiling effect. Such ceiling effects have already been observed in previous research (45, 77). This result is in line with the observed tendency of shorter dish names (78).

## Data availability statement

The raw data supporting the conclusions of this article will be made available by the authors, without undue reservation.

## Ethics statement

The studies involving human participants were reviewed and approved by Institutional Review Board, Modul University Vienna. The patients/participants provided their written informed consent to participate in this study.

## Author contributions

MG initiated the conceptualization of the project, wrote the theoretical part of the manuscript, collected data, conducted analyses, and was responsible for the structure and content of the manuscript. EW wrote the empirical part of the manuscript, collected data, and conducted analyses. JH contributed to the conceptualization of the project, designed the empirical study, and contributed to the theoretical part of the manuscript. All authors contributed to the article and approved the submitted version.

## Funding

Open Access Funding provided by University of Applied Sciences Wiener Neustadt.

## Conflict of interest

The authors declare that the research was conducted in the absence of any commercial or financial relationships that could be construed as a potential conflict of interest.

## Publisher’s note

All claims expressed in this article are solely those of the authors and do not necessarily represent those of their affiliated organizations, or those of the publisher, the editors and the reviewers. Any product that may be evaluated in this article, or claim that may be made by its manufacturer, is not guaranteed or endorsed by the publisher.
